# The Usefulness of Serum Vitamin D Levels in the Assessment of IBD Activity and Response to Biologics

**DOI:** 10.3390/nu13020323

**Published:** 2021-01-22

**Authors:** Marco Valvano, Marco Magistroni, Antonio Mancusi, Debora D’Ascenzo, Salvatore Longo, Gianpiero Stefanelli, Filippo Vernia, Angelo Viscido, Stefano Necozione, Giovanni Latella

**Affiliations:** 1 Gastroenterology, Hepatology and Nutrition Division, Department of Life, Health and Environmental Sciences, University of L’Aquila, 67100 L’Aquila, Italy; valvano.marco@libero.it (M.V.); magistroni.marco@gmail.com (M.M.); a.mancusi8@gmail.com (A.M.); debora.dascenzo97@gmail.com (D.D.); salvator.longo@gmail.com (S.L.); giastefanelli@gmail.com (G.S.); filippo.vernia1@gmail.com (F.V.); angelo.viscido@univaq.it (A.V.); 2 Epidemiology Unit, Department of Life, Health and Environmental Sciences, University of L’Aquila, 67100 L’Aquila, Italy; stefano.necozione@univaq.it

**Keywords:** inflammatory bowel disease, Crohn’s disease, ulcerative colitis, vitamin D, biological therapy

## Abstract

The main role of vitamin D is calcium homeostasis and bone metabolism, although its activity as an immuno-modulator and its anti-inflammatory effect is well-known. Low blood vitamin D levels are common among patients with inflammatory bowel disease (IBD). Whether low vitamin D levels could affect the disease activity or it is an effect of a worse condition of the disease is still unclear. This study aimed to investigate the role of blood vitamin D levels to identify the clinical, endoscopic, and histological activity in a cohort of patients with ulcerative colitis (UC) or Crohn’s disease (CD) on therapy with biological drugs. In this retrospective cohort study, 50 IBD patients (24 UC and 26 CD) that underwent colonoscopy from January 2017 to January 2020 with a concomitant serological evaluation of vitamin D were included. Patients with clinical, endoscopic, and histological activity and those who lost their clinical response to the biological drug had lower vitamin D levels compared to patients in remission or patients that did not change therapeutic regimens. A receiver operating characteristic (ROC) analysis and Youden’s Index were performed to assess the optimal vitamin D levels to identify patients with the active disease. The ROC analysis showed an area under the curve (AUC) of 0.709 (*p* = 0.005; confidence interval (CI): 0.564–0.829), 0.769 (*p* < 0.001; CI: 0.628–0.876), and 0.810 (*p* < 0.001; CI: 0.670–0.910) for the clinical, endoscopic, and histological outcomes, respectively. The optimal vitamin D cut-off was ≤25 ng/mL. The vitamin D level is an additional useful tool in the evaluation of IBD patients with good accuracy to predict their endoscopic and histological activity and clinical response to biologics.

## 1. Introduction

Inflammatory bowel diseases (IBD), which include ulcerative colitis (UC) and Crohn’s disease (CD), are chronic relapsing diseases [1,2]. Typically, the IBD clinical course is characterized by the alternation of periods of remission and flare-ups [3]. Although the etiology of IBD is still unclear, it involves a complex interaction between the genetic, environmental, and microbial factors and the immune responses [4].

Several studies have shown that vitamin D contributes to maintenance gut epithelial barrier integrity against inflammatory and pathogenic stimuli, suggesting that vitamin D plays a protective role in the pathogenesis of IBD [5,6]. The main role of vitamin D is calcium homeostasis and bone metabolism, although several potential actions have been suggested in the last two decades [7]. In particular, vitamin D deficiency has been reported in several chronic diseases associated with increased inflammation and deregulation of the immune system, including IBD [8,9]. It is estimated that 13% and 10% of CD and UC patients, respectively, have a calcium deficiency [10]. It is important to underline that the prevalence of osteopenia or osteoporosis is higher among IBD patients on corticosteroid therapy [11], although, among these patients, low vitamin D levels and bone disease were observed independently of glucocorticoid administration [10]. A systematic review including 14 observational studies assessed the prevalence of vitamin D deficiency. It was 31.6% in UC and 38.1% in CD [12].

Among IBD patients, whether low vitamin D levels could affect the disease activity or if it is a secondary effect of a worse condition of the disease is still unclear [13].

A recent meta-analysis showed that, among IBD patients, low vitamin D levels were linked with increased odds of disease activity (odds ratio (OR) 1.53, 95% confidence interval (CI) 1.32–1.77, *I*^2^ = 0%), mucosal inflammation (OR 1.25, 95% CI 1.06–1.47, *I*^2^ = 0%), low quality of life (QOL) scores (OR 1.30, 95% CI 1.06–1.60, *I*^2^ = 0%), and clinical relapse (OR 1.23, 95% CI 1.03–1.47, *I*^2^ = 0%) [14]. It is important to underline that the definition of low vitamin status is extremely variable among the studies included in this meta-analysis, ranging from 10 to 35 ng/mL [15,16]. The optimal cut-off of serum vitamin D levels for the evaluation of disease activity in IBD patients has not been defined. Therefore, the vitamin D level as a tool for the evaluation of disease activity in IBD patients is far from being adopted.

The aim of this study was to investigate the efficacy of blood vitamin D levels to identify clinical, endoscopic, and histological activity in a cohort of patients with UC or CD on therapy with biological drugs.

## 2. Materials and Methods

### 2.1. Study Population

This is a retrospective, single-center, cohort study including IBD patients followed at the Gastroenterology, Hepatology and Nutrition division of the University of L’Aquila (L’Aquila, Italy).

All patients gave written consent to the anonymous processing of their data. Ethics approval was issued by the Internal Review Board of the University of L’Aquila (Protocol number: 9029). All clinical investigations were conducted according to the principles laid down in the Declaration of Helsinki.

IBD patients on biological therapy that underwent colonoscopy from January 2017 to January 2020 with a concomitant serological evaluation of vitamin D (within two months and without therapeutic change) were included. The diagnoses of UC and CD were based on standard clinical, endoscopic, histological, and cross-sectional imaging criteria [17]. All CD patients (always at diagnosis but, also, during follow-up when clinically needed) underwent routine cross-sectional imaging techniques, such as transabdominal ultrasound, computed tomography, or magnetic resonance, to evaluate in a more precise and complete way the localization, extension, and activity of their intestinal lesions.

Demographic (gender and age) and clinical data (years from diagnosis, IBD-related medications, oral supplementation of vitamin D, and season for sample collection) were collected.

### 2.2. Outcomes

Our primary outcome was the assessment of the optimal vitamin D cut-off (25-OH-D concentration in the serum) to identify patients with the active disease.

A clinical flare-up was defined by a clinical score. Partial Mayo clinic scores (PMS) and the Harvey-Bradshaw Index (HBI) were adopted for UC and CD, respectively. PMS ≥ 2 in UC and HBI ≥ 5 in CD identified the clinical activity [18].

Each patient was categorized with a Mayo endoscopic score (MES) or a simple endoscopic score (SES-CD) for UC and CD, respectively, based on the overall appearance of the ileal and colonic mucosa. MES ≥ 2 or SES-CD ≥ 3 corresponded to endoscopic activity [18].

At least two biopsies were taken from the ileum and all colon segments (cecum, ascending colon, transverse colon, descending colon, and sigmoid colon) and rectum for the pathological examination. The endoscopy biopsies were sectioned and stained with hematoxylin and eosin for pathological examination. The histological activity was defined by the presence of inflammatory cell infiltrate in at least one of the histological sections [19].

Loss of response (LOR) to biological therapy was defined as the reappearance of significant and quantifiable clinical signs, e.g., moderate–severe activity evaluated with a specific clinical score (PMS and HBI for UC and CD, respectively), steroid dependence or steroid-refractory disease, and the need to change the biological drug treatment [20,21].

### 2.3. Vitamin D Serum Concentration Assay

An automated chemiluminescence system was used to determine the vitamin D (25-OH-D) concentrations in the serum (LIAISON^®^ 25 OH Vitamin D TOTAL Assay 310600—DiaSorin Inc., Stillwater, MN, USA).

### 2.4. Statistical Analysis

For all statistical analyses, SAS software (version 9.4, Copyright© 2021–2012 by SAS Institute Inc., Cary, NC, USA) and MedCalc software (version 19.6 MedCalc^®^ Ltd., Ostend, Belgium) were used. Continuous variables were reported as means with standard deviations (±SD). An unpaired two-sample *t*-test was performed to compare the vitamin D levels among active and inactive patients for each group (clinic, histologic, and endoscopic).

A receiver operating characteristic (ROC) analysis and Youden’s Index were performed to assess the accuracy of the vitamin D level evaluations to identify patients with the active disease for each group (clinic, endoscopic, and histologic activities) and the optimal vitamin D cut-off.

A McNemar test was performed to compare patients with endoscopic or histologic activity and identify those with the optimal vitamin D cut-off and patients with clinical activity.

A logistic regression test was used to evaluate the association, expressed as the odds ratio (OR), between vitamin D levels lower than the optimal cut-off and disease activity.

## 3. Results

Fifty IBD patients in biological therapy (24 UC and 26 CD) who underwent a colonoscopy from January 2017 to January 2020 in our unit, with a concomitant serological evaluation of vitamin D, were included in our analysis. All patients included in the study were Italian.

The blood samples for the vitamin D assessment were collected within two months before or after the colonoscopy with biopsies.

Overall, 17/50 (34%) patients presented clinical activity, 27/50 (54%) and 31/47 (65.9%) patients showed endoscopic and histological activity, respectively. Among the patients in clinical remission, 13/33 (39.4%) and 16/31 (51.6%) showed endoscopic and histological activity, respectively. Finally, 6/50 (12%) lost their response to therapy with biological drugs.

The baseline characteristics are shown in Table 1.

**Figure 1:**
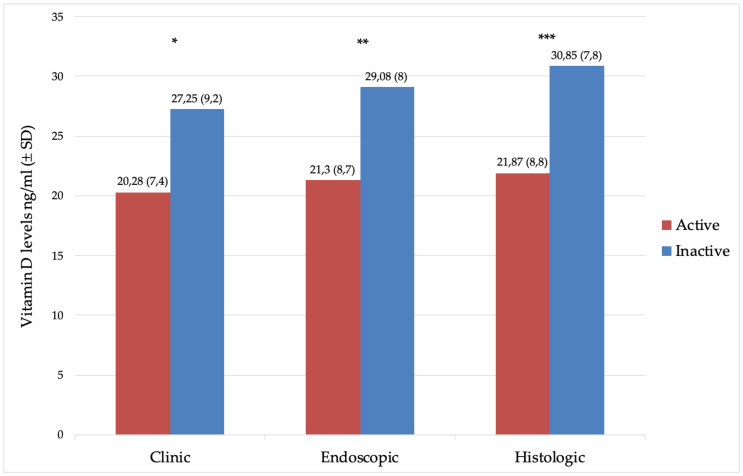
Mean levels (±SD) of vitamin D (ng/mL) in patients with the active and inactive disease. (*) *p* = 0.0062, (**) *p* = 0.002, and (***) *p* = 0.0013.

Patients with clinical, endoscopic, and histological activities had lower vitamin D levels compared to patients in remission (Figure 1).

### 3.1. ROC Analysis and Optimal Cut-Off

**Figure 2:**
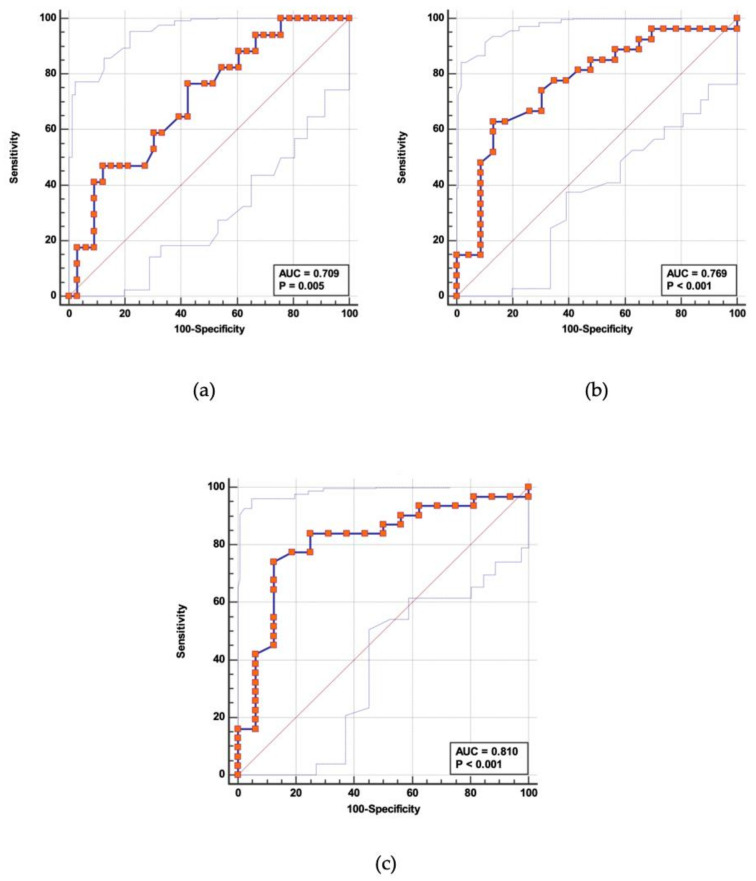
The receiver operating characteristic (ROC) analysis to set the optimal cut-off value of vitamin D to assess the (**a**) clinical activity, (**b**) endoscopic activity, and (**c**) histologic activity. AUC: area under the curve.

The ROC analysis and Youden’s Index were performed to assess the optimal vitamin D levels to identify patients with the active disease. The ROC analysis showed an area under the curve (AUC) of 0.709 (*p* = 0.005; CI: 0.564–0.829), 0.769 (*p* < 0.001; CI: 0.628–0.876), and 0.810 (*p* < 0.001; CI: 0.670–0.910) for the clinical, endoscopic, and histological outcomes, respectively (Figure 2).

**Figure 3:**
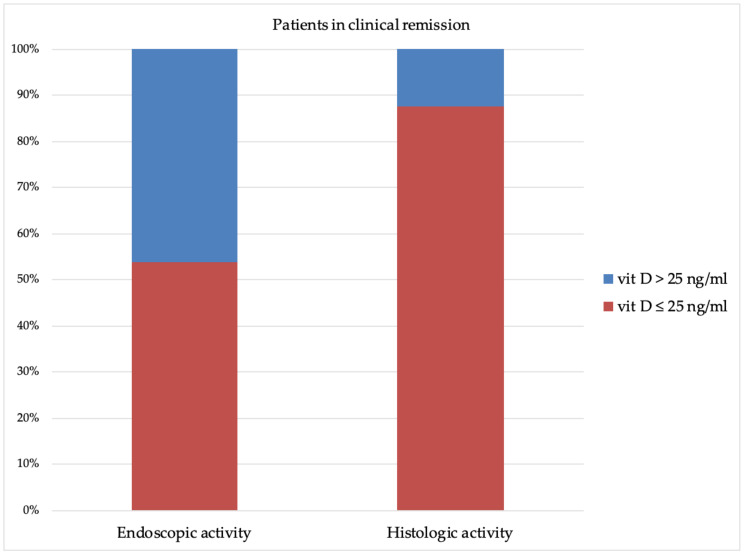
Vitamin D levels in patients in clinical remission and endoscopic or histologic activity.

For the AUC with better performance (histologic group; AUC 0.810), the Youden’s Index assessed the optimal vitamin D cut-off at ≤25 ng/mL. It showed the best accuracy and identified 53.8% (7/13) and 87.5% (14/16) of the patients as having endoscopic and histologic activity, although these patients were in clinical remission (McNemar’s test: *p* = 0.0184) (Figure 3). Furthermore, vitamin D ≤25 ng/mL identified 100% (6/6) of the patients who lost their response to biological drugs (6/50).

### 3.2. Vitamin D under the Set Threshold and Odds Ratio of Disease Activity

A logistic regression test was used to evaluate the association between vitamin D levels lower than the optimal cut-off and disease activity. The odds ratio (OR) was 6.44 (CI: 1.7–26.5) for clinical activity, OR 11.33 (CI: 2.7–47.9) for endoscopic activity, and OR 20.12 (CI: 3.7–108.6) for histologic activity.

## 4. Discussion

Despite the evidence of the associations between disease activity and low vitamin D levels [14], its usefulness in the assessment of IBD patients is debated [22,23,24]. A plethora of biomarkers with various accuracies have been proposed for the evaluation of the disease’s activity in IBD patients [25,26]. However, the usefulness of serum vitamin D levels in the assessment of disease activity in an IBD population has not been carried out. Furthermore, the optimum vitamin D cut-off has not yet been established to assess the real risk of clinical relapse [23].

Moreover, if low vitamin D levels could affect the disease activity or if it is a secondary effect of a worse condition of the disease is still unclear [13]. It was reported that adult IBD patients had higher odds of vitamin D deficiency when compared to healthy controls (OR = 1.81; 95% CI: 1.37, 2.40; *I*^2^ = 1%; *p* < 0.0001) [12].

In CD, the ileal localization of lesions or surgical resection of the distal ileum may lead to bile acid diarrhea and contribute, with the use of bile acid sequestrants, to the vitamin D deficiency. However, in our cohort of CD patients, the rate of both ileal and/or ileocolic involvement of the lesions and ileal surgery was not significantly different between patients with the active disease and patients with the inactive disease (Table 1). Moreover, the CD patients with an inactive disease more often showed an ileal localization of the disease. Therefore, the disease activity appears to play an important role in inducing a vitamin D deficiency. An inverse correlation between CD clinical activity and the blood levels of vitamin D were found (*r* = −0.42276; *p* = 0.0314)

A randomized clinical trial showed that the relapse rate among patients treated with a supplementation of vitamin D was lower (6/46 or 13%) compared to a placebo (14/48 or 29%), and the survival analysis showed higher relapse-free time in the group of patients treated with vitamin D supplementation (hazard ratio (HR), 0.42; CI 0.16–1.10) (*p* = 0.06) [27].

A single-center observational study evaluated if the vitamin D status before antitumor necrosis factor-α (anti-TNFα) therapy influenced the durability of treatment in IBD patients. This studied showed an inverse association with the durability of anti-TNFα treatment and vitamin D levels, with a more pronounced effect on patients with CD. Patients with insufficient vitamin D showed an early cessation of anti-TNFα therapy due to a loss of response (HR, 3.49; 95% CI, 1.34–9.09) [28].

In addition, a five-year observational study including 965 IBD patients showed that patients with a low mean vitamin D level required more steroid, biologics, emergency department visits, hospital admissions, and surgery compared with subjects with normal mean vitamin D levels (*p* < 0.05) [29].

Several evidences suggested a potential role of vitamin D in the pathophysiology of IBD, although further studies are warranted to clarify this issue.

The primary outcome of our study was to assess the optimal vitamin D levels to identify the endoscopic or histologic active disease and to determine the usefulness of serum vitamin D levels in IBD patients with both the clinical active and inactive disease.

The ROC analysis has shown good accuracy of the vitamin D serum level, under a set threshold, to identify patients with endoscopic or histological activity. In particular, using as the optimal cut-off ≤ 25 ng/mL, the sensitivity and specificity were 74–88% and 74–70% in the assessment of histologic and endoscopic activity, respectively. The AUC was 0.810 (*p* = 0.001; CI:0.670–0.910) and 0.769 (*p* < 0.001; CI: 0.628–0.876) for the histologic and endoscopic activity evaluations, respectively.

Our results showed lower vitamin D levels in patients with the active disease (expressed as clinical, endoscopic, or histologic activity). Moreover, adopting the cut-off established with the ROC analysis and the Youden’s index, insufficient vitamin D levels were linked with increased odds of disease activity.

Furthermore, among patients who stopped treatment due to a loss of response (6/50), vitamin D levels ≤ 25 ng/mL were observed in all cases.

Our study had some limitations. In particular, the small sample size was the main limitation, and larger studies are needed to confirm our results. Second, the observational design made it difficult to assess a cause-effect ratio between the low vitamin D status and disease activity. On the other hand, the use of a rigid score for the evaluation of our outcome made our results reliable.

In conclusion, taking into account the widespread use of vitamin D estimation for a global assessment in IBD patients, its low cost, and the accuracy of its evaluation for the assessment of the disease activity, we suggested that vitamin D may be used to predict endoscopic and histologic activity or a loss of response of biological therapy in IBD patients. The optimal cut-off was identified at ≤25 ng/mL.

The vitamin D level is an additional useful tool in the evaluation of IBD patients, with sufficient accuracy to predict the endoscopic and histological activity.

## Figures and Tables

**Table 1:** Baseline demographic and clinical characteristics.

	Clinical Evaluation (*n* = 50)		Endoscopic Evaluation (*n* = 50)		Histologic Evaluation (*n* = 47)	
	Active (17)	Inactive (33)	Active (27)	Inactive (23)	Active (31)	Inactive (16)
Male/female	10/7	16/17	16/11	10/13	18/13	8/8
Age	52 (22–76)	51.5 (21–76)	51.5 (21–65)	52.5 (28–76)	52 (21–65)	51.5 (28–76)
Years from diagnosis	12.5 (1–28)	13 (1.2–36)	12.5 (1–33)	13 (1.2–36)	13.5 (12–36)	12.5 (1.2–34)
Crohn’s Disease (CD)	7 (41%)	19 (58%)	14 (52%)	12 (52%)	15 (48%)	9 (56%)
CD localization						
Ileal	1 (14%)	9 (47%)	4 (29%)	6 (50%)	5 (33%)	5 (55%)
Colonic	1 (14%)	0 (0%)	1 (7%)	0 (0%)	1 (7%)	0 (%)
Ileo-colonic	5 (72%)	10 (53%)	9 (64%)	6 (50%)	9 (60%)	4 (45%)
Ulcerative Colitis (UC)	10 (59%)	14 (42%)	13 (48%)	11 (48%)	16 (52%)	7 (44%)
UC localization						
Proctitis	0 (0%)	0 (0%)	0 (0%)	0 (0%)	0 (0%)	0 (0%)
Left sided colitis	7 (70%)	5 (36%)	8 (62%)	4 (36%)	9 (56%)	3 (43%)
Pancolitis	3 (30%)	9 (64%)	5 (38%)	7 (64%)	7 (44%)	4 (67%)
Intestinal surgery						
CD	4 (57%)	11 (58%)	7 (50%)	8 (67%)	9 (60%)	5 (56%)
UC	0 (0%)	0 (0%)	0 (0%)	0 (0%)	0 (0%)	0 (0%)
Therapy						
Infliximab	4 (23%)	12 (36%)	8 (30%)	8 (35%)	9 (29%)	6 (37%)
Adalimumab	3 (18%)	7 (21%)	6 (22%)	4 (17%)	6 (19%)	2 (13%)
Vedolizumab	5 (29%)	14 (43%)	8 (30%)	11 (47%)	11 (36%)	8 (50%)
Golimumab	2 (12%)	0 (0%)	2 (7%)	0 (0%)	2 (6%)	0 (0%)
Ustekinumab	3 (18%)	0 (0%)	3 (11%)	0 (0%)	3 (10%)	0 (0%)
Oral supplementation Vitamin D						
Yes	7 (41%)	17 (52%)	11 (41%)	13 (57%)	14 (45%)	8 (50%)
No	8 (47%)	15 (45%)	14 (52%)	9 (39%)	15 (48%)	7 (44%)
n.a.	2 (12%)	1 (3%)	2 (7%)	1 (4%)	2 (7%)	1 (6%)
Season of sample collection						
Autumn	4 (24%)	3 (9%)	4 (15%)	3 (14%)	5 (16%)	2 (13%)
Winter	6 (35%)	10 (30%)	9 (33%)	7 (30%)	8 (26%)	5 (31%)
Spring	3 (17%)	9 (27%)	6 (22%)	6 (26%)	9 (29%)	3 (19%)
Summer	4 (24%)	11 (33%)	8 (30%)	7 (30%)	9 (29%)	6 (37%)

*n* (Median; range); *n* (%). n.a.: not applicable. CD: Chron’s disease. UC: ulcerative colitis.

## Data Availability

No supporting data is available.
